# Hyperbaric Oxygen Therapy for the Treatment of Bone-Related Diseases

**DOI:** 10.3390/ijms26031067

**Published:** 2025-01-26

**Authors:** Jie Feng, Chenyu Zhu, Jun Zou, Lingli Zhang

**Affiliations:** 1 School of Exercise and Health, Shanghai University of Sport, Shanghai 200438, China; fjtuotuo0113@163.com (J.F.); zhuchenyu156@163.com (C.Z.); zoujun@sus.edu.cn (J.Z.); 2 College of Athletic Performance, Shanghai University of Sport, Shanghai 200438, China

**Keywords:** hyperbaric oxygen therapy, bone, bone formation, bone resorption

## Abstract

Hyperbaric oxygen therapy (HBOT) is a therapeutic modality that enhances tissue oxygenation by delivering 100% oxygen at pressures greater than 1 absolute atmosphere. In recent years, HBOT has shown considerable potential in the treatment of bone diseases. While excess oxygen was once thought to induce oxidative stress, recent studies indicate that when administered within safe limits, HBOT can notably promote bone healing and repair. Extensive basic research has demonstrated that HBOT can stimulate the proliferation and differentiation of osteoblasts and encourage bone angiogenesis. Furthermore, HBOT has been shown to exert a beneficial influence on bone metabolism by modulating the inflammatory response and redox status. These mechanisms are closely related to core issues of bone biology. Specifically, in the context of fracture healing, bone defect repair, and conditions such as osteoporosis, HBOT targets the key bone signaling pathways involved in bone health, thereby exerting a therapeutic effect. Several clinical studies have demonstrated the efficacy of HBOT in improving bone health. However, the optimal HBOT regimen for treating various bone diseases still requires further definition to expand the indications for its clinical application. This paper outlines the mechanisms of HBOT, focusing on its antioxidant stress, promotion of bone vascularization, and anti-inflammatory properties. The paper also describes the application of HBOT in orthopedic diseases, thereby providing a scientific basis for the development of precise and personalized HBOT treatment regimens in clinical orthopedics.

## 1. Introduction

Oxygen is an essential element for maintaining the normal physiological functions of the human body. As a crucial component of the tissue microenvironment, oxygen is transported throughout the body via gas media (air and airways) and liquid media (blood), playing a vital role in the body’s metabolism [1]. Bone is a highly vascularized connective tissue. Oxygen, along with nutrients, hormones, neurotransmitters, and growth factors, is delivered to bone cells through the bone’s vascular system [2]. Additionally, blood circulation is closely linked to bone remodeling processes through the interaction of osteoblasts, osteocytes, osteoclasts, and vascular cells [3]. Oxygen in the blood also plays a key role in bone metabolism, serving as an essential metabolic substrate for enzymatic reactions in the body [4]. Therefore, increasing attention has been directed toward understanding the role of oxygen in bone biology and the effects of variations in oxygen partial pressure on skeletal tissues. Within this framework, hyperbaric oxygen (HBO) therapy has emerged as a focal point of investigation. HBO refers to an artificially induced environment characterized by the administration of high concentrations of oxygen under pressures exceeding atmospheric levels [5]. Its distinctive physiological effects not only elucidate the pivotal role of oxygen in regulating the bone microenvironment but also provide a promising research direction for the development of innovative therapeutic strategies for orthopedic diseases.

**Figure 1:**
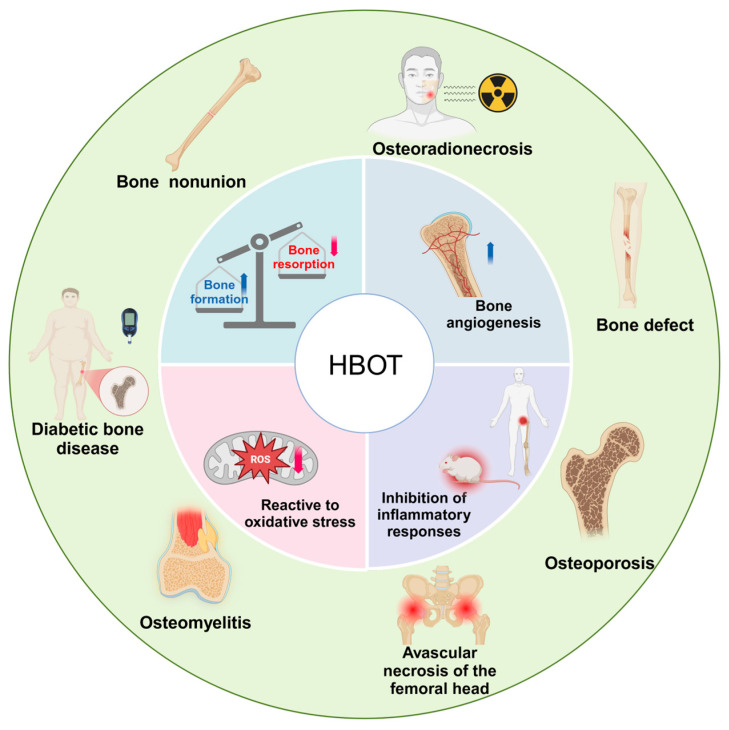
Mechanisms underlying the therapeutic effects of HBOT in bone-related disorders. HBOT enhances bone formation, inhibits resorption, promotes vascularization, mitigates oxidative stress, and suppresses inflammation. These effects collectively support its efficacy in treating conditions such as osteoporosis, diabetic bone disease, bone defects, Bone nonunion, osteoradionecrosis, avascular necrosis of the femoral head, and osteomyelitis.

With the rising demand for orthopedic treatments, particularly for complex conditions like fractures and bone defects, exploring novel therapies has become imperative. Hyperbaric oxygen therapy (HBOT) is a non-invasive technique based on HBO conditions, where patients breathe 100% oxygen intermittently at pressures exceeding 1 absolute atmosphere [6]. The increase in air pressure enhances the delivery of oxygen to deep tissues by improving the diffusion gradient. The dissolved oxygen produced by HBOT has a range of physiological effects, including but not limited to increasing plasma soluble oxygen levels, enhancing immune responsiveness, promoting new collagen deposition, and stimulating angiogenesis, thereby altering the tissue’s response to disease and injury [7,8,9]. Over the past two decades, HBOT has been widely used in the treatment of clinical bone diseases, either as a standalone or adjuvant therapy, because of its regulatory effects on the bone microenvironment [10,11]. However, to further enhance the clinical efficacy of HBOT, it is crucial to elucidate its underlying mechanisms in key physiological effects, including antioxidation, promotion of bone angiogenesis, and anti-inflammatory actions. This review aims to explore the core mechanisms of HBOT and its clinical applications in orthopedic diseases, providing theoretical support and practical guidance for its precise therapeutic use (Figure 1).

## 2. Mechanism of HBOT on the Bone

### 2.1. HBOT Promotes Bone Formation and Inhibits Bone Resorption

The maintenance of bone homeostasis relies on the dynamic balance between bone formation and resorption, with osteoblasts and osteoclasts playing central roles in this process. Osteoblasts are primarily derived from mesenchymal stem cells in the periosteum and bone marrow. Their functions include synthesizing and secreting the bone matrix, participating in the formation and maintenance of bone tissue, and regulating osteoclast activity through cell–cell contact, cytokines, and the extracellular matrix, thereby ensuring bone homeostasis [12,13,14]. Osteoclasts are hematopoietic cells derived from granulocyte–macrophage colony-forming units, which branch off from the monocyte–macrophage lineage early in differentiation [15]. Osteoclasts degrade mineralized bone matrix to release minerals, such as calcium and phosphorus, into the bloodstream, thereby regulating the renewal and remodeling of bone tissue. Their differentiation and activity are controlled by the OPG/RANK/RANKL pathway, working in synergy with osteoblasts to maintain a balance in bone metabolism [16,17].

HBOT regulates bone metabolism through multiple pathways and holds remarkable potential for maintaining bone homeostasis and treating bone diseases. HBOT promotes osteoblast proliferation and differentiation via the FGF-2/MEK/ERK/Akt/p70/NF-κB and PKC/JNK signaling pathways, enhancing bone mineralization [18,19,20]. Additionally, HBOT can reduce the surface area of osteoclasts and lower sclerostin levels, thereby mitigating bone loss [21].

Simultaneously, HBOT may maintain bone metabolic balance by regulating the OPG/RANK/RANKL pathway. On the one hand, HBOT can improve ANFH by upregulating serum OPG levels. OPG prevents its interaction with RANK by binding to RANKL, thus maintaining the balance between OPG/RANK/RANKL and enhancing its antagonistic effect on RANKL [22], which is crucial for bone homeostasis [23]. On the other hand, HBOT suppresses osteoclast formation and bone resorption by downregulating RANK levels [24,25]. In a rat model of ovariectomy, HBOT and combined exercise therapy substantially reduced femoral RANKL levels and effectively alleviated bone loss induced by ovariectomy [26]. In normal weight and obese rats, HBOT effectively reversed the age-related changes in RANKL and RANK levels, while also improving the damage to the bone microstructure caused by aging [27].

Additionally, HBOT further regulates bone homeostasis by inhibiting the expression of hypoxia-inducible factor (HIF) mRNA and protein [24]. HIF, a key transcription factor in hypoxic environments, plays a crucial role in the metabolic reprogramming of osteoblasts to support bone formation under anaerobic conditions [28,29]. HBOT reduces the levels of HIF-1α and HIF-2α, improves hypoxia-related metabolic disorders, and increases adenosine triphosphate levels, thereby accelerating bone formation and restoring bone mineralization capacity [27,30].

**Figure 2:**
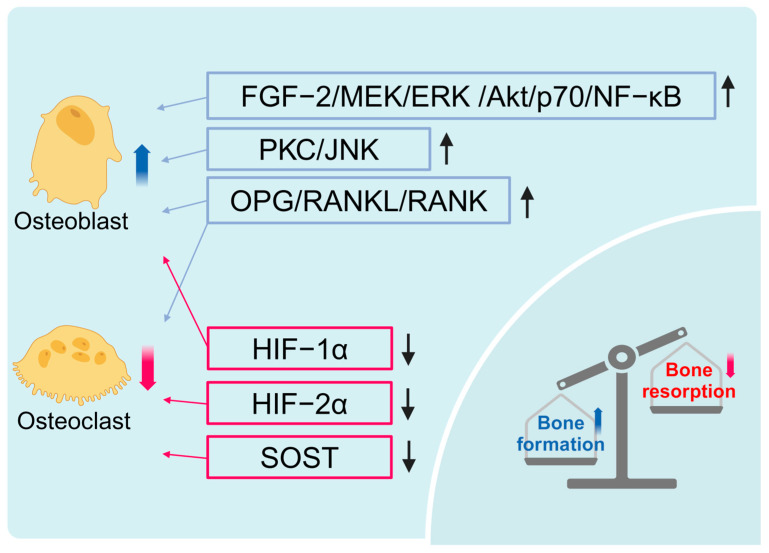
Molecular mechanisms of HBOT in bone homeostasis regulation. HBOT maintains bone homeostasis by balancing osteoblast-mediated bone formation and osteoclast-mediated resorption. It enhances osteoblast proliferation, differentiation, and mineralization through the FGF-2/MEK/ERK, Akt/p70/NF-κB, and PKC/JNK signaling pathways. HBOT also modulates the OPG/RANK/RANKL axis, increasing OPG’s antagonistic effect on RANKL to promote bone formation, while downregulating RANK to inhibit osteoclastogenesis and resorption. Furthermore, HBOT reduces HIF-1α to promote bone formation and suppresses HIF-2α to inhibit bone, resorption.

Therefore, HBOT promotes bone formation by stimulating osteoblast proliferation and mineralization through various pathways that regulate bone formation and resorption. HBOT also inhibits osteoclast formation and bone resorption by regulating the OPG/RANK/RANKL pathway. Additionally, HBOT reduces HIF levels, improves hypoxia-related metabolic disorders, and further enhances bone microstructure (Figure 2).

### 2.2. HBOT Promotes Bone Vascularization

The highly developed vascular network in bones receives approximately 10% to 15% of the resting cardiac output, and maintaining its health is critical for the survival and function of bone cells [31,32]. During skeletal development in mammals, bone formation is closely linked to the growth and formation of blood vessels [33]. In addition to supplying oxygen and nutrients, blood vessels serve as the basic building blocks of bone units, transporting hormones and progenitor cells to support bone remodeling, and providing a pathway for immune cells to enter and exit the bone marrow [32,34]. Oxygen is essential for angiogenesis, which is, to some extent, dose-dependent [35]. HBOT has considerable potential to promote bone vascularization by effectively increasing plasma soluble oxygen levels, thereby enhancing the diffusion gradient of oxygen delivery to deep tissues [7].

In particular, HBOT has been demonstrated to effectively promote the angiogenic response and bone formation. In a rat femoral diaphysis defect model, a single daily dose of HBOT was observed to accelerate bone repair and vascular ingrowth compared to the control group [36]. Furthermore, in a rabbit mandible traction model, HBOT markedly altered the osteogenic pattern of irradiated bone, enhancing vascular density. This effect was particularly evident in animals that were not irradiated and received HBOT [37]. Similarly, in a canine mandibular traction defect model, the trabecular bone density and bone area in the HBOT group were substantially higher than those in the control group. Moreover, remarkable bone regeneration and angiogenesis were observed at the tension site [38].

Furthermore, HBOT has been shown to enhance bone formation by facilitating osteogenic–angiogenic coupling. Endothelial cells, the primary components of the blood vessel wall, secrete angiogenic factors that interact with those released by osteoblasts, thereby coordinating the angiogenic and osteogenic processes [39]. In a mouse craniotomy model, HBOT notably increased the bone volume around the reattached skull flap and the volume of H-shaped blood vessels, while promoting the proliferation of mature osteoblasts and endothelial cells. Further mechanistic studies revealed that, after HBOT treatment, the expression of genes related to adhesion and migration was upregulated, and cell–cell interactions were enhanced, particularly in the angiogenesis factor 2 and integrin α5β1 pathway signals [40]. In a rat skull defect model, the HBOT group exhibited a substantial increase in the number of CD31-positive endothelial marker cells at week 2, along with an upregulation of bone morphogenetic protein (BMP) levels and a notable downregulation of the pro-inflammatory cytokines IL-1 and IL-6. By week eight, the HBOT group showed remarkable improvements in bone healing and vascularization [41]. Additionally, HBOT reduced damage to endothelial activity in tibial blood vessels caused by smoke exposure, thereby improving delayed bone healing [42,43]. Specifically, ANFH leads to elevated serum levels of S100 calcium-binding protein A9 (S100A9), which induces endothelial cell apoptosis and arterial dysfunction [44,45]. Proteomic analysis revealed that HBOT reduced serum levels of S100A9 in ANFH patients, thereby promoting angiogenesis, which may be one of the mechanisms through which HBOT improves bone healing in ANFH patients [46].

Additionally, HBOT upregulated the expression of vascular endothelial growth factor (VEGF) in rabbit skull defect tissue, thereby promoting bone healing [47]. The four- and eight-week HBOT upregulated VEGF and BMP-2 levels in dog alveolar sockets, effectively simulating early bone formation, mineralization, and reconstruction in extraction sockets [48]. VEGF, a highly specific peptide growth factor, not only regulates angiogenesis and its maintenance but also plays a key role in coordinating osteogenesis and angiogenesis. This phenomenon is realized through the coupling of intrachondral and intramembranous bone formation and acting as a downstream factor of HIF-1α during skeletal development [46,49,50]. The regulation of VEGF expression by HBOT amplifies its role in enhancing bone healing.

**Figure 3:**
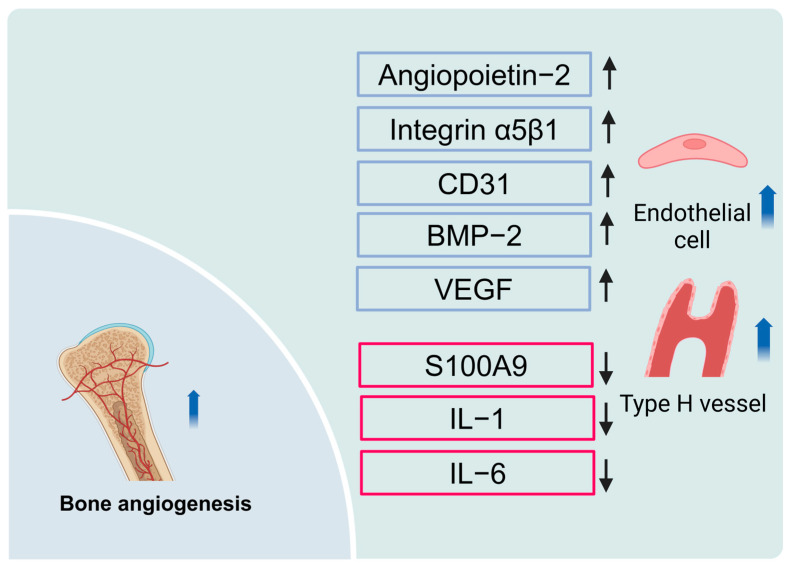
Molecular mechanisms of HBOT in bone healing and angiogenesis. HBOT significantly upregulates the expression of VEGF and BMP-2, promoting early bone formation, mineralization, and bone remodeling. Additionally, HBOT enhances bone volume and type H vessel formation around the cranial bone graft in mice, stimulating the proliferation of mature osteoblasts and endothelial cells via the angiopoietin-2 and integrin α5β1 signaling pathways. HBOT also downregulates the elevated expression of S100A9 associated with ANFH, reducing endothelial cell apoptosis and improving vascular function. Moreover, HBOT decreases pro-inflammatory cytokines IL-1 and IL-6, leading to a significant increase in CD31-positive endothelial cells and creating an anti-inflammatory microenvironment conducive to bone regeneration.

HBOT has generally been demonstrated to enhance angiogenesis and oxygen supply, while optimizing the bone microenvironment by facilitating interactions between osteoblasts and endothelial cells. This synergistic effect not only promotes bone healing and regeneration but also provides a mechanistic basis and potential clinical applications for the repair of bone injuries (Figure 3).

### 2.3. HBOT Reduces Oxidative Stress

Oxidative stress refers to a dysregulation in the intracellular redox balance that occurs when the production of reactive oxygen species (ROS) exceeds the scavenging capacity of the antioxidant system [51]. ROS are inevitable by-products of cellular oxygen metabolism, primarily generated by the leakage of electrons from the mitochondrial electron transport chain during oxidative phosphorylation [52]. Normal levels of ROS play a crucial role in initiating various biological processes, such as apoptosis, survival, and differentiation, by activating signaling pathways that function as second messengers [53,54]. In the context of bone remodeling, the physiological redox state is particularly important [55]. Excessive ROS, as an endogenous mediator of osteoclast differentiation, leads to bone loss by promoting the differentiation of osteoclast precursor cells and inhibiting osteoblast activity and bone formation function [56,57,58].

HBOT has been demonstrated to regulate the redox state, enhance the antioxidant capacity of the body, and play a crucial role in tissue repair and anti-aging [11,59]. Two cycles of HBOT (30 cycles of 90 min each) resulted in an initial increase in plasma ROS levels in 23 patients with ischemic necrosis of the femoral head. However, these levels regressed after 30 treatments, accompanied by a reduction in inflammatory markers and a decrease in edema observed on imaging. This finding indicates that HBOT may provide preconditioning protection by enhancing the endogenous antioxidant capacity of the body [60]. The ROS generated by short-term HBOT are regarded as signaling molecules that promote a reduction in mitochondrial activity, leading to decreased ROS production and, consequently mitigating oxidative stress [61]. In contrast to the hypothesis that ROS accumulation and enhanced osteoclast activity would occur, HBOT effectively improved D-galactose-induced bone microstructural changes and partially mitigated the negative effects of obesity on bone metabolism in a premature aging rat model [62]. Additionally, in a rat osteomyelitis model, long-term HBOT resulted in lower serum levels of malondialdehyde, superoxide dismutase (SOD), and glutathione peroxidase compared to the model group. Additionally, HBOT effectively alleviated the symptoms of osteomyelitis in these rats [63]. Therefore, the long-term effects of HBOT differ from those of short-term treatment, being characterized by a reduction in oxidative stress and inflammation, which subsequently leads to improved bone pathology [64,65,66]. However, the precise dose of HBOT needed to elicit antioxidant effects remains uncertain. This uncertainty is due to the need to achieve an optimal balance between preventing excessive accumulation of free radicals and enhancing mitochondrial activity, thereby strengthening antioxidant defenses [8].

**Figure 4:**
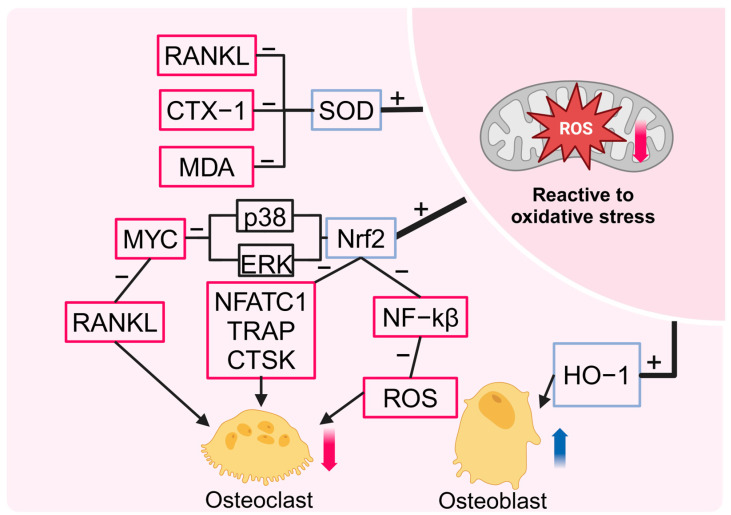
Redox regulation mechanisms of HBOT in bone homeostasis and repair. HBOT significantly improves bone homeostasis and repair by modulating the redox state. First, HBOT upregulates SOD levels, inhibiting the expression of RANKL, CTX-1, and MDA, thereby alleviating bone loss. Second, HBOT activates the Nrf2 and its downstream pathways, suppressing MYC expression and reducing RANKL levels. Additionally, through the upregulation of Nrf2, HBOT inhibits the expression of NFATc1, TRAP, and Ctsk, further suppressing osteoclast activity. Moreover, the upregulation of Nrf2 reduces NF-κB activity, mitigates ROS accumulation, and inhibits osteoclast differentiation and function. Concurrently, HBOT enhances HO-1 expression, promoting osteoblast activity and accelerating bone formation and repair.

From a mechanistic perspective, HBOT has been demonstrated to induce the expression of antioxidant enzymes and promote oxidative defense through the activation of nuclear factor erythroid 2-related factor 2 (NRF2) and its downstream signaling pathways [67]. NRF2 regulates the gene expression of various antioxidant enzymes by binding to the antioxidant response element (ARE), playing a crucial therapeutic role in conditions such as kidney injury [68], brain injury [69,70,71,72], lung injury [73,74], and diabetic foot ulcers [75]. Notably, NRF2 has also demonstrated considerable potential in the regulation of metabolic bone diseases, including osteoporosis and Paget’s disease [76,77]. Specifically, the transcription factor NRF2 has been demonstrated to regulate osteoclast differentiation by modulating intracellular ROS signaling [78]. Overexpression of NRF2 enhances the increases in antioxidant enzyme levels induced by RANK ligand, while simultaneously inhibiting osteoclast differentiation [79]. Conversely, inhibition of NRF2 leads to a reduction in antioxidant enzyme expression, promoting increased osteoclast activity [80,81]. In the early stages of osteoclast differentiation, NRF2, acts as an upstream regulator of myelocytomatosis transcription factors, negatively regulating osteoclastogenesis induced by RANKL by mediating the transcriptional repression of MYC via the ERK and p38 signaling pathways [82]. Additionally, NRF2 inhibits NF-κB activity and directly binds to the c-Fos promoter, reducing ROS accumulation and the phosphorylation of c-Fos, which effectively suppresses osteoclast formation during the early stages of RANK signaling [83,84]. In the final stage of osteoclast differentiation, NRF2 exerts its inhibitory effect on osteoclast function by reducing the levels of NFATc1, tartrate-resistant acid phosphatase, and Ctsk [85,86]. Heme oxygenase-1 (HO-1), a key downstream signaling molecule of NRF2, plays an important role in maintaining bone homeostasis by regulating osteoblasts and osteoclasts [87]. Notably, exposure to HBO at 4ATA and 37 °C for 4 h markedly elevates DNA damage in osteoblasts, accompanied by elevated HO-1 levels. This evidence indicates that HBOT induces an antioxidant response in osteoblasts [88]. This finding aligns with the preceding assertion that ROS may function as preconditioning signaling molecules in HBOT, activating intracellular signaling pathways that provide adaptive protection against long-term oxidative stress [61]. Indeed, HO-1 has been demonstrated to play an adaptive protective role in lymphocytes [89], spinal neurons [90], and the liver [91]. This evidence indicates that HO-1 is a key factor in the antioxidant response to HBOT, potentially preventing early tissue damage. Furthermore, HBOT has been demonstrated to be crucial in preventing and repairing bone loss by regulating oxidative stress levels. For instance, the combination of HBOT and treadmill exercise has been shown to upregulate serum levels of SOD in ovariectomized rats. This phenomenon is accompanied by a reduction in the expression of RANKL and CTX-1 mRNA, which subsequently leads to an improvement in the deterioration of bone microstructure and a reduction in bone loss [26]. In an aged, obese rat model, HBOT was observed to increase the expression levels of Sirt1 and CuZnSOD, effectively alleviating oxidative stress and promoting the balance of bone metabolism [27]. In an open fracture model, HBOT was observed to reduce the inflammatory response and reperfusion injury by elevating SOD levels and decreasing malondialdehyde levels. Additionally, HBOT has been demonstrated to promote osteoblast proliferation and accelerate fracture healing [92]. As a key antioxidant enzyme, SOD plays a critical role in maintaining bone homeostasis. The regulatory role of SOD is evident in its promotion of osteogenic differentiation and inhibition of adipogenic differentiation in mesenchymal stem cells, mediated through the PI3K/AKT and MAPK pathways [93]. Furthermore, SOD has been shown to reduce bone loss by inhibiting osteoclast differentiation and alleviating oxidative stress [94]. The upregulation of SOD levels by HBOT further reinforces its crucial role in maintaining bone metabolism balance and supporting bone repair (Figure 4).

### 2.4. HBOT Effectively Suppresses Inflammation

The role of immune cells in the bone microenvironment is becoming increasingly recognized. Damage to the bone tissue elicits an immune response, initially mediated by neutrophils, mast cells, monocytes and macrophages, followed by the involvement of the adaptive immune system (e.g., T and B cells). The onset of the inflammatory response facilitates the recruitment and activation of immune cells, such as macrophages, T cells, and B cells, within the bone microenvironment. These cells contribute to the remodeling and repair of bone tissue by releasing cytokines and chemokines [95,96]. Macrophages play a dual role in this process. On the one hand, they promote inflammation by secreting pro-inflammatory factors, including TNF-α, IL-1β, and IL-6. On the other hand, macrophages also mediate bone tissue repair by releasing anti-inflammatory factors such as IL-4, IL-10, and IL-13, and various growth factors [97,98]. However, prolonged or chronic expression of inflammatory molecules has been demonstrated to negatively affect skeletal health. Excessive production of pro-inflammatory factors not only directly inhibits osteoblast activity but also stimulates the production and activation of osteoclasts, leading to increased bone resorption [99].

HBOT has been shown to regulate immune cell function and improve bone changes induced by inflammation, thereby exhibiting a substantial anti-inflammatory effect. Gardin et al. [100] demonstrated that human adipose-derived stem cells (hADSCs) from individuals undergoing HBOT in an inflammatory environment exhibited notably enhanced osteogenic differentiation. The cells had substantially higher levels of calcium deposition in the extracellular matrix by day 14 compared to the control group. This finding indicates that HBOT not only facilitates tissue repair through its anti-inflammatory effects but may also directly influence the osteogenic capacity of mesenchymal stem cells by modulating stress and oxygen signals. However, by day 21, the level of calcium deposition in cells treated with hyperbaric pressure and hyperoxia decreased, and the rate of late-stage proliferation was reduced, indicating that while stress and oxygen signals can promote osteogenic differentiation, they also have a limiting effect. Additionally, the anti-inflammatory impact of HBOT may depend on the dosage and duration of treatment.

**Figure 5:**
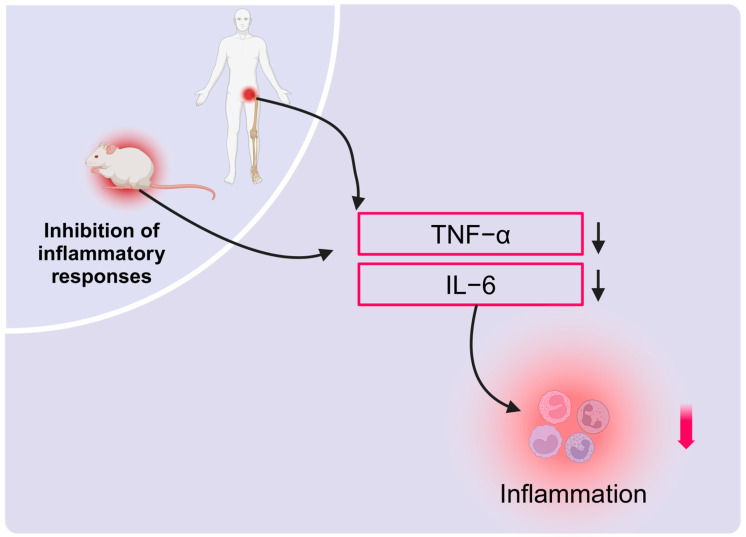
Modulation of the bone microenvironment by HBOT through anti-inflammatory mechanisms. HBOT significantly reduces pro-inflammatory cytokines TNF-α and IL-6, exerting a positive effect on the bone microenvironment. In the early stages of AVNFH, HBOT lowers plasma TNF-α and IL-6 levels, demonstrating its anti-inflammatory action. In obesity and premature aging models, HBOT reduces plasma TNF-α and IL-6 levels, preventing age-related bone loss.

Furthermore, HBOT has been shown to exert a beneficial impact on the bone microenvironment, particularly through a marked reduction in pro-inflammatory factors such as tumor necrosis factor-alpha (TNF-α) and interleukin-6 (IL-6). In the early stages of AVNFH, HBOT was observed to markedly diminish plasma levels of TNF-α and IL-6 in patients, thereby demonstrating a discernible anti-inflammatory effect [60]. Similarly, in rats with obesity and premature aging, HBOT has been demonstrated to restore abnormal levels of TNF-α and IL-6, effectively preventing age-related bone loss [27]. The results of these studies collectively demonstrate that HBOT regulates the bone microenvironment through its anti-inflammatory properties. Moreover, HBOT plays a crucial role in supporting bone tissue repair and maintaining metabolic balance by enhancing immune cell function (Figure 5).

## 3. HBOT in Clinical Skeletal Diseases

The aforementioned mechanisms have sparked considerable interest in the potential of HBOT for treating clinical orthopedic diseases over the past two decades. This potential has been extensively explored through animal experimentation. This review analyzed the existing literature on the application of HBOT in the treatment of orthopedic diseases (Table 1).

### 3.1. Osteoporosis

Osteoporosis is a systemic bone disease induced by an imbalance between bone formation and resorption. This imbalance leads to a reduction in bone mass, destruction of the bone tissue microstructure, and increased brittleness [101]. Recent studies have demonstrated that HBOT can effectively counteract degenerative changes in the skeleton in physiological and pathological states as a non-drug treatment.

Mild HBOT (1317 hPa, 40% oxygen concentration) has effectively improved cortical and trabecular bone loss due to caudal suspension. This improvement is achieved by reducing the osteoclast surface area and sclerostin levels [21]. Similarly, in a model of disuse osteoporosis induced by spinal cord injury, ultra-early HBOT has been shown to slow down bone loss by enhancing the synthesis of calcitonin gene-related peptide in sensory neurons in the dorsal horn of the spinal cord, which consequently promotes bone formation and inhibits bone resorption [102].

Transient osteoporosis of the hip (TOH) is a distinct pathological condition that differs from metabolic osteoporosis. This condition primarily affects young and middle-aged women and men, with a particularly high prevalence during the third trimester of pregnancy and the postpartum period. The condition is characterized by bone marrow edema in the femoral head and neck, leading to localized demineralization and dysfunction [103]. HBOT has been demonstrated to aid recovery from TOH by reducing intraosseous edema, promoting vasoconstriction, and stimulating bone repair [104]. Furthermore, HBOT has been found to be an effective intervention for alleviating pain and improving hip joint mobility in individuals with TOH [105,106].

In a rat model of premature aging by D-galactose, elevated levels of advanced glycation end products (AGEs) are associated with increased inflammation, oxidative stress, impaired bone remodeling, and reduced bone strength, further exacerbated by obesity. HBOT has been demonstrated to have an anti-osteoporosis effect, notably enhancing the bone microstructure in lean senescent rats and partially restoring bone homeostasis in those with obesity-related bone disorders [27,62]. Thus, obesity may influence the therapeutic outcomes of HBOT. On one hand, obesity is typically associated with chronic low-grade inflammation, metabolic dysregulation, and increased oxidative stress in adipose tissue, all of which may attenuate the beneficial effects of HBOT on the bone microenvironment [107,108]. On the other hand, alterations in hemodynamic parameters in obese individuals could further limit the efficacy of HBOT [109]. This variability in treatment outcomes underscores the critical need for standardized treatment protocols.

Therefore, HBOT, as an innovative non-drug treatment, has shown considerable potential in managing osteoporosis and related conditions. However, despite these encouraging preliminary results, further clinical trials are necessary to confirm its long-term efficacy and establish optimal treatment protocols. With continued research, HBOT may offer new approaches to skeletal health management, but its effectiveness and safety must be further explored and validated across diverse populations.

### 3.2. Diabetic Bone Disease

Bone damage and an increased risk of fractures are common complications in type 1 (T1D) and type 2 (T2D) diabetes [110]. Hyperglycemia substantially inhibits proliferation and induces apoptosis of osteoblasts through the accumulation of AGEs, ultimately weakening bone strength [111,112]. Additionally, certain antidiabetic drugs (such as thiazolidinediones) can activate peroxisome proliferator-activated receptor gamma (PPARγ) in bone marrow cells, further contributing to bone loss and an elevated fracture risk [113]. In this context, HBOT, as a non-invasive therapy, holds potential for improving diabetic bone health by promoting osteogenesis.

The beneficial effects of HBOT on diabetic osteopathy has been corroborated in numerous animal models. In a rat model of diabetes, P. Limirio et al. [65] showed that T1D reduces collagen maturation and mineral deposition, thereby weakening the biomechanical properties of the skeleton. However, exposure to HBOT at 2.5 ATA every 48 h substantially increased the maximum strength and stiffness of the femur in diabetic rats. Subsequent studies indicated that osteogenesis was markedly impaired in the diabetic group compared to the nondiabetic group, but HBOT drastically improved early bone regeneration in diabetic rats [114]. These findings indicate that the osteogenic effects of HBOT are closely related to its promotion of bone angiogenesis. In another study, rats with T1D were treated with daily HBOT for 90 min over five consecutive days at 2.5 ATA. This treatment resulted in a notable increase in the area of newly formed bone, along with remarkable improvements in endothelial cell proliferation and the density of intrabony microvessels [115]. Furthermore, HBOT has been demonstrated to facilitate early healing of implant osseointegration in a diabetic rabbit model, providing additional histological and biomechanical evidence of its osteogenic potential [116].

Therefore, HBOT has been demonstrated to be an effective treatment for alleviating bone loss caused by hyperglycemia. This condition is achieved through its capability to improve the bone microenvironment, promote angiogenesis, and enhance bone strength. Additionally, HBOT has been demonstrated to markedly accelerate bone regeneration, showing considerable promise in the treatment of diabetic bone disease.

### 3.3. Bone Defect

A bone defect refers to a pathological condition in which bone tissue is partially or completely absent due to factors such as trauma, infection, or tumor resection. This condition can have substantial impacts on the functional abilities and quality of life of patients [117]. While conventional treatments such as autologous bone grafting and synthetic bone substitutes have been proven effective, they are frequently associated with complications, such as issues at the donor and graft sites, as well as suboptimal repair outcomes [118]. In this context, HBOT has emerged as a promising adjuvant treatment for bone defect healing. By increasing the partial pressure of oxygen, HBOT has been demonstrated to accelerate tissue repair, promote bone healing, and stimulate angiogenesis [119].

The fundamental principle of bone defect repair involves the synthesis and remodeling of the bone matrix. Studies have shown that HBOT can facilitate effective bone regeneration. In several studies on postoperative healing in rabbit skull defect models, HBOT was observed to markedly enhance bone regeneration at the defect site [120]. Additionally, HBOT may accelerate bone healing by enhancing the resorption rate of residual graft material at the defect site following autologous bone grafting [119,121]. In rodents, HBOT has been demonstrated to upregulate Runx2 expression levels during the early stage of femoral defect healing (three days after surgery). Moreover, HBOT has been shown to reduce postoperative inflammation, thereby accelerating bone healing [122]. Similar results were observed in a rat model of diabetic bone defects, where HBOT effectively enhanced new bone formation at the defect site during the early postoperative period (day 7) [114]. Additionally, a recent study revealed a link between HBOT and osteoblast activity. A nine-day course of HBOT stimulated the Piezo1-YAP pathway in osteoblasts, inducing bone formation and promoting osteogenic–vascular coupling during bone repair. This osteogenic effect, similar to mechanical simulation, contributed to improved healing of bone defects in mice [123].

Notably, the formation of bone is contingent upon the presence of blood vessels [124]. HBOT has been demonstrated to stimulate the proliferation and migration of endothelial cells, thereby promoting the formation of new blood vessels by increasing oxygen concentration in tissues [125]. In a rat model of a femoral diaphysis defect, HBOT considerably accelerated endochondrial ossification and bone repair with daily treatment [36]. Additionally, growth factors play a crucial role in bone angiogenesis facilitated by HBOT. In a rabbit skull model, six weeks of HBOT resulted in increased VEGF expression in the tissue, compared to treatment with normal oxygen. This effect persisted for two weeks after the completion of treatment [47]. Similarly, in a mouse model of cranial defects, HBOT at two weeks post-surgery stimulated angiogenesis in the periosteum of the regenerating bone region and upregulated the expression of basic fibroblast growth factor (bFGF) in young and adult mice, thereby accelerating bone regeneration [126].

Therefore, HBOT has been demonstrated to enhance bone formation and angiogenesis, making it a promising adjunctive treatment for bone defect healing. Further research should concentrate on the long-term effects and underlying mechanisms of HBOT, as well as its potential for combination with other treatments to further advance bone defect repair. Additionally, exploring the use of adaptive HBOT for treating different types of bone defects and specific patient populations will be essential in optimizing clinical outcomes and developing personalized treatment strategies.

### 3.4. Bone Nonunion

A fracture is classified as a nonunion when it fails to heal within nine months of the original injury due to factors such as inadequate fixation, insufficient blood supply, or bacterial infection, and shows no signs of progressive bone healing for three consecutive months [127]. Recent reports indicate an increased incidence of nonunion in simple and complex diaphyseal fractures from 2015 to the present. Nonunion occurs in approximately 7–10% of long bone fractures treated surgically [128]. The current treatment options primarily involve bone grafting and the use of bone biological agents [129,130]. However, only a limited number of studies have investigated the role of HBOT. In one study on infectious nonunion of the tibia, 2 out of 14 patients developed reinfection following radical debridement, fixation, osteotomy and callus traction, and antibiotic therapy. Subsequent HBOT was found to effectively control the infection [131]. One additional study examined the combined effects of platelet-rich plasma and HBOT on aseptic tibial nonunion. In this study, 20 HBOT sessions over four weeks, along with the Ilizarov technique, resulted in accelerated bone healing, although functional outcomes remained unchanged in the patients [132]. Furthermore, after 20 consecutive days of HBOT, the experimental group showed a marked increase in osteogenic activity by day 90 following rabbit atrophic nonunion [133]. However, the current evidence remains limited due to reliance on a single data source and a lack of direct, more clinically-oriented evidence. Further research is required to confirm the efficacy of HBOT in treating bone nonunion.

### 3.5. Osteoradionecrosis

Osteoradionecrosis (ORN) is a notable complication resulting from radiation therapy used to treat malignant neoplasms, particularly in the head and neck region [134]. Prolonged exposure to high doses of ionizing radiation (typically exceeding 50 Gy) can induce tissue hypoxia and vascular degeneration, which subsequently impairs the proliferative capacity of bone marrow, collagen, and endothelial cells. This phenomenon ultimately leads to the involution of facial bones and necrosis of the overlying soft tissues [135,136,137]. Preliminary studies have shown that HBOT is effective throughout the entire disease cycle of ORN, possibly due to its distinctive oxygen-based mechanisms and effects [138]. However, as relevant clinical research has progressed, the actual efficacy of HBOT in treating ORN has become a topic of growing debate. Therefore, this section presents the most recent research findings on the use of HBOT for ORN treatment and analyzes potential reasons for the observed discrepancies in its efficacy evaluation.

The origins of HBOT in the context of ORN date back to 1983, when Marx et al. first proposed its use. Subsequent studies have extensively explored the potential of HBOT to promote angiogenesis, collagen synthesis, and bone regeneration by alleviating hypoxia and enhancing tissue regeneration [139,140,141]. Based on these mechanisms, HBOT was initially considered a standard adjuvant treatment for ORN. Several clinical studies have demonstrated that preoperative HBOT can markedly enhance wound healing in patients [142]. For example, Korambayil et al. [143] reported a case of an elderly patient with ORN who underwent 28 sessions of HBOT. Following treatment, a notable improvement was observed in the oxygenation of the mandibular tissue, and the surgical incision healed without complications. Additionally, animal studies have highlighted the osteogenic and angiogenic effects of HBOT. In a rabbit model of mandibular radiation injury, 18 preoperative HBOT sessions (2.5 atmospheres, 90 min each) resulted in notable improvements in osteogenic patterns and enhanced angiogenesis [37].

Despite initial support for the therapeutic potential of HBOT in treating ORN, recent clinical guidelines and numerous studies have raised concerns about its widespread use. The latest guidelines from the International Society of Oral Oncology-Multinational Association for Supportive Care in Cancer acknowledge that while HBOT may offer some therapeutic benefits, its general applicability in preventing and managing ORN remains controversial due to the limitations in the current quality of evidence [144]. For example, discrepancies in study design, inconsistent application of the ORN classification system, and variability in HBOT treatment protocols collectively contributed to the challenges in establishing reliable conclusions from existing research [145]. In a multicenter trial conducted by Forner et al. [146], 65 patients with ORN were followed for one year after surgery. The findings indicated that HBOT did not notably improve the healing of radiation necrosis subsequent to surgical excision when compared to conventional treatment approaches. Furthermore, notable discrepancies in patient responses to HBOT were observed. A systematic review of the literature over the past 30 years recommends that HBOT should not be used routinely in the absence of sufficient evidence. Rather, HBOT should be considered an adjunctive treatment in high-risk cases or when conservative treatments have failed [147]. At the same time, the timing of therapeutic intervention significantly influences the outcomes of ORN. Evidence indicates that HBOT should primarily be considered for early-stage ORN, while definitive surgical treatment is more suitable for advanced or refractory cases [148]. A retrospective case–control study has further emphasized that surgical intervention should be the preferred treatment for patients with advanced ORN. Relying solely on HBOT or delaying surgical intervention may lead to incomplete healing and other adverse sequelae, particularly in stage III ORN. Moreover, treatment selection must consider several crucial factors, including the patient’s general health condition (such as nutritional status and age), the specific treatment goals, and the disease’s stage [149].

The discrepancy in efficacy assessments may be closely associated with the incomplete understanding of the pathogenesis of ORN. The “radiation–hypoxia–nonhealing” theory, originally proposed by Marx, posits that radiation-induced local hypoxia and ischemia result in metabolic imbalance and chronic wound healing disorders, thereby triggering ORN [139]. However, recent research has gradually redefined ORN as a fibrosing atrophic disease, emphasizing the roles of free radical generation, inflammatory responses, and microvascular thrombosis in its development of following radiation [150]. These recent findings have elevated the role of antifibrotic therapy in the management of ORN, with theoretical advancements influencing the selection of treatment strategies. Therefore, further investigation of the optimal use of HBOT in combination with other therapies, such as growth factors and stem cell therapies, is necessary.

While the full potential of HBOT in the treatment of ORN remains to be fully elucidated, HBOT may offer a valuable adjunctive therapeutic option for high-risk patients by improving the local microenvironment and facilitating tissue repair. Further research should concentrate on clarifying the pathological mechanisms of ORN, optimizing the therapeutic parameters of HBOT, and investigating its combination with innovative treatment modalities to enhance patient outcomes and facilitate the advancement of precision medicine.

### 3.6. Avascular Necrosis of the Femoral Head

In contrast to the mechanism of ORN, ANFH represents the death of bone tissue due to interruption of the blood supply. This condition is frequently associated with factors such as trauma, steroid use, or alcohol abuse [151]. The pathological features of ANFH include apoptosis of osteocytes and osteoblasts, which are induced by ischemia. This process leads to femoral head collapse and cartilage degeneration, ultimately resulting in the development of secondary osteoarthritis [152]. HBOT has recently garnered considerable attention for its potential to improve functional outcomes and prognosis in patients with ANFH.

The initial pathological process of ANFH is primarily characterized by ischemia and inflammation. This inflammatory state leads to edema in the necrotic area, joint pain, and cartilage degeneration [153]. Core decompression (CD) is considered the gold standard for treating ANFH because it can substantially joint pressure and pain [154,155]. Clinical evidence shows that HBOT can achieve similar therapeutic effects to CD, particularly in the early stages of the disease. A retrospective cohort study analyzed 23 patients (12 treated with CD and 11 with HBOT) and found that 66.7% of the CD group and 81.8% of the HBOT group experienced satisfactory improvement in hip function, confirming the effectiveness of HBOT in the early treatment of ANFH [156]. Furthermore, Bozkurt et al. [157] conducted a comparative analysis of HBOT alone and HBOT combined with CD. Their findings indicated that both treatments improved pain and functional scores, with the combined treatment offering potentially enhanced efficacy. A reasonable assumption is that this therapeutic effect is closely related to the regulatory effects of HBOT on inflammation and ROS. A study involving 23 male patients demonstrated that two cycles of HBOT, comprising 30 sessions of 90 min each, led to a notable reduction in TNF-α and IL-6 levels [60]. Similarly, another study by the same research team found that HBOT not only effectively alleviates pain in ANFH patients but also increases serum OPG levels, which subsequently promotes bone regeneration through the OPG/RANK/RANKL pathway [22].

The long-term pathological difficulties associated with ANFH can be attributed to insufficient blood supply and the presence of bone defects, which are major obstacles in repairing ischemic bone tissue necrosis. Proteomics analysis indicates that HBOT treatment can reduce the serum S100A9 levels in patients with ANFH, thereby mitigating its inhibitory effect on angiogenesis [46]. Furthermore, a study using a multilayer perfusion imaging model demonstrated that HBOT positively influences the hemodynamic state in ANFH. In this model, regional blood flow and blood volume were significantly lower in the model group compared to the control group. However, HBOT effectively increased regional blood volume and mean transit time [158]. These effects are likely attributed to the oxygen-rich environment created by HBOT, which promotes the synthesis of growth factors, enhances wound healing, and reduces post-ischemic and post-inflammatory damage [159]. These findings were further supported by animal model studies. In a rat femoral head necrosis model, HBOT notably increased tissue oxygen tension, enhanced the activity of fibroblasts, angiogenic cells, and osteoblasts, and accelerated the repair of necrotic bone [160].

Although HBOT does not offer a fundamental cure for femoral head necrosis, it has shown considerable potential in improving functional outcomes and patient prognosis. By alleviating ischemia, modulating inflammatory responses, and stimulating bone tissue regeneration, HBOT provides substantial benefits, even in the absence of a definitive cure. These effects position HBOT as an important adjunctive therapy, leading to notable improvements in pain relief and functional recovery. Future research should aim to refine HBOT treatment parameters and explore its potential synergistic effects with other interventions, thereby enhancing its clinical application and supporting the development of personalized treatment strategies.

### 3.7. Osteomyelitis

Osteomyelitis is a destructive bone tissue disease caused by pathogenic microorganisms, primarily affecting the cortical and periosteal tissues. Most cases present arise following bone surgery or as a consequence of vascular insufficiency [161]. The treatment of bone and joint infections is particularly challenging due to limited penetration of antimicrobials into bone tissue, which is relatively avascular, as well as the presence of drug-resistant microorganisms [162,163,164]. Given the inhibitory effects of HBOT on anaerobic organisms and its potential to promote vascular proliferation, researchers are beginning to focus on the benefits of HBOT for the treatment of osteomyelitis [165]. This review explores the role of HBOT in managing osteomyelitis, with a particular focus on its antimicrobial effects.

The antimicrobial effects of HBOT can be divided into direct and indirect effects, both of which are closely related to its underlying mechanism. Chronic osteomyelitis often develops due to the formation of bacterial biofilms, which not only increase pathogen resistance to antibiotics but also continue to trigger a local inflammatory response, exacerbating the chronicity of the disease [166]. Studies have shown that HBOT enhances the antibiotic efficiency against bacterial biofilms and reduces bacterial resistance, making it particularly valuable in the treatment of refractory osteomyelitis [167]. Available evidence indicates that HBOT demonstrates antimicrobial efficiency comparable to that of cephalosporin and gentamicin, with an excellent stacking effect when used in combination [168,169,170]. Additionally, HBOT has been demonstrated to enhance antibiotic penetration into bone tissue, overcoming the limitations posed by the relatively poor blood supply in bone [171]. By increasing the oxygen content in infected bone tissue, HBOT also restores leukocyte functionality, improving their capability to eliminate Gram-positive bacteria (e.g., Staphylococcus aureus) [172]. The results of clinical trials provide further support the antimicrobial effects of HBOT in treating osteomyelitis. Bingham et al. [173] treated 28 patients with refractory osteomyelitis with HBOT, in which the source of infection included Staphylococcus aureus, Klebsiella group, Pseudomonas aeruginosa, Escherichia coli, and Enterobacteriaceae, most often in mixed infections. The results demonstrated that at 2 atmospheres for 2 h, 5 days a week, HBOT facilitated the recovery of all patients, with no recurrence over the following 6 years. A recent study of 80 patients with chronic refractory osteomyelitis of the foot revealed that 68 patients treated with a similar regimen (2.5 atmospheres, 5 days a week, 2 h each time) were completely cleared of the infection and remained relapse-free during a three-year follow-up period [174].

Overall, HBOT demonstrated remarkable clinical value in the treatment of refractory osteomyelitis, as evidenced by its capability to enhance the efficacy of antibiotics, inhibit bacterial biofilm formation, and restore the local oxygenation status. With the improvement in understanding of HBOT mechanisms and the accumulation of clinical experience, more optimized HBOT treatment regimens may emerge in the future. These advancements could notably enhance its application in treating osteomyelitis, particularly in addressing the growing challenge of drug-resistant bacterial infections.

**Table 1:** Dosage and indications for the use of HBOT in clinical skeletal disorders.

Species	Time	Frequency	Pressure (bar)	Duration (min)	Adaptation Disease	Reference
Mouse	60	5 days/week	2.5	90	Cranial bone defects	[126]
Rat	14	Once per day	2	80	Osteoporosis	[62]
Rat	40	Twice daily (Initial course) Once daily (Subsequent courses)	2.2	40	Osteoporosis	[102]
Rat	15	Once every two days	2.5	90	Type 1 diabetic bone disease	[65]
Rat	15	5 days/week	2.4	90	Type 1 diabetic bone disease	[115]
Rat	1–7	Once per day	2.5	90	Femoral bone defect	[122]
Rat	7	Once per day	2.5	90	Diabetes mellitus combined with femoral bone defects	[114]
Rat	20	5 days/week	2	90	Femoral bone defect	[36]
Rat	2–42	Once per day	2.5	90	Ischemic necrosis of the femoral head	[160]
Rabbit	10	Once per day	2.4	90	Diabetic Bone Disease Implant Integration	[116]
Rabbit	20	Once per day	2.4	90	Cranial bone defects	[120]
Rabbit	15–30	5 days/week	2.4	90	Radial stem bone defect	[119]
Rabbit	20	5 days/week	2.4	90	Cranial bone defects	[47]
Rabbit	20	Once per day	2.5	120	Atrophic tibial nonunion	[133]
Rabbit	18	Once per day	2.5	90	Traction osteogenesis of the irradiated mandible	[37]
Rabbit	30	Once per day	2.0	45	Ischemic necrosis of the femoral head	[158]
Human	40, 90	5 days/week	2.5	90	Transient osteoporosis of the hip joint	[105]
Human	20	Once per day	2.5	90	Aseptic tibial nonunion	[132]
Human	30–60 (determined based on the course of the disease)	5 days/week	2.4	90	Radiation-induced osteonecrosis and irradiation-induced wounds	[143]
Human	20–40	3–4 times/week	2.2	90	Ischemic necrosis of the femoral head	[156]
Human	30	6 days/week	2.4	120	Osteonecrosis of the hip	[157]
Human	60 (2 cycles)	Once a day, with a 30-day interval between weeks	2.5	90	Ischemic necrosis of the femoral head	[60]
Human	60 (2 cycles)	5 days/week	2.4	90	Ischemic necrosis of the femoral head	[22]
Human	Treatment is stopped in the presence of signs of recovery and the condition has clearly stopped for two weeks	5 days/week	2	120	Refractory osteomyelitis	[173]
Human	50	5 days/week	2.5	120	Refractory osteomyelitis	[174]

## 4. Limitations: Adverse Events in HBOT

Despite the encouraging clinical outcomes associated with HBOT, the occurrence of adverse events highlights the necessity for the establishment of standardized protocols for its application. The most commonly reported adverse events include barotrauma, pulmonary complications, neurological symptoms, and ophthalmic complications [175,176,177,178]. Barotrauma can include middle ear damage (e.g., congestion or ruptured eardrum), sinus pain, and toothache, typically caused by gas expansion [175]. Pulmonary complications may arise from gas retention or alveolar rupture due to pulmonary hyperpressure, with more severe cases increasing the risk of mediastinal emphysema and pneumothorax [179,180]. Additionally, considering whether medical devices implanted in patients (such as pacemakers) have undergone stress testing is necessary to ensure their safety during treatment [181]. Elevated oxygen levels can lead to pulmonary oxygen toxicity, manifested as coughing and respiratory distress. Prolonged exposure to high concentrations of oxygen may also trigger neurological toxicity, with symptoms including tunnel vision, tinnitus, nausea, muscle twitching, and, in rare cases, seizures. These symptoms usually resolve rapidly once oxygen exposure is discontinued [182,183,184]. Ophthalmic complications may include retinopathy, cataracts, and transient myopic changes. While these complications are considered reversible, the resolution period is estimated to be several months to a year [178,185]. Therefore, prior to the implementation of HBOT, one recommendation is to conduct comprehensive screening of patients to ascertain their suitability for treatment and identify any potential risks. Furthermore, enhancing the monitoring and management of patients and providing suitable assistance and intervention during treatment are pivotal strategies for mitigating the occurrence of adverse events.

## 5. Conclusions

HBOT has demonstrated considerable potential in the clinical treatment of bone diseases, primarily due to its capability to exert a range of mechanisms. On the cellular level, HBOT has been shown to promote the proliferation and differentiation of osteoblasts and vascular endothelial cells, thereby enhancing bone formation and bone vascularization. At the molecular level, HBOT helps maintain the equilibrium of bone reconstruction processes by reducing oxidative stress, regulating the release of inflammatory mediators, and modulating signaling pathways such as OPG/RANK/RANKL. These combined mechanisms provide a strong rationale for the potential application of HBOT in bone repair and regeneration.

Future research is expected to explore the combination of HBOT with advanced technologies, such as biomaterials and stem cell therapy, as regenerative medicine and tissue engineering continue to evolve. This approach holds the potential to offer more effective treatment options for complex bone defects and bone diseases. Additionally, the emergence of personalized medicine has introduced novel avenues for the use of HBOT. Tailoring treatment plans to address the unique pathological conditions of individual patients will help optimize the efficacy of HBOT. Simultaneously, further large-scale multicenter clinical trials and fundamental research are required in the future to comprehensively investigate the underlying mechanism of HBOT and the safety and efficacy of its clinical application. This approach will facilitate the establishment of a robust scientific foundation for the comprehensive treatment of bone diseases.
